# Reappraise the Situation but Express Your Emotions: Impact of Emotion Regulation Strategies on *ad libitum* Food Intake

**DOI:** 10.3389/fpsyg.2012.00359

**Published:** 2012-09-25

**Authors:** Diana Taut, Britta Renner, Adriana Baban

**Affiliations:** ^1^Department of Psychology, Babeş-Bolyai University of Cluj-NapocaCluj-Napoca, Romania; ^2^Department of Psychology, University of KonstanzKonstanz, Germany

**Keywords:** emotion regulation, food intake, suppression, reappraisal, emotional eating

## Abstract

Research investigating the role of maladaptive emotion regulation (ER) on food intake has exclusively focused on food intake in a forced consumption situation. In contrast, the present study examined the effect of negative emotions (fear, negative affect) and ER strategies (suppression, reappraisal) on food intake in a non-forced, free eating setting where participants (*N* = 165) could choose whether and how much they ate. This free (*ad libitum*) eating approach enabled, for the first time, the testing of (1) whether eating (yes/no) is used as a secondary ER strategy and (2) whether the amount of food intake differed, depending on the ER strategy. In order to produce a more ecologically valid design, ER strategy manipulation was realized while exposing participants to emotion induction procedures. To induce an initial negative emotional state, a movie clip was presented without ER instruction. The instructions to regulate emotions (suppression, reappraisal, no ER instruction) then preceded a second clip. The results show that whereas about two-thirds of the control (no ER instruction) and suppression groups began to eat, only one-third of the reappraisal group did. However, when reappraisers began to eat, they ate as much as participants in the suppression and control groups. Accordingly, the results suggest that when people are confronted with a negative event, eating is used as a secondary coping strategy when the enacted ER is ineffective. Conversely, an adaptive ER such as reappraisal decreases the likelihood of eating in the first place, even when ER is employed during rather than before the unfolding of the negative event. Consequently, the way we deal with negative emotions might be more relevant for explaining emotional eating than the distress itself.

## Introduction

The relationship between negative emotions and eating has been extensively researched over the years. Current knowledge about the impact of negative emotions on eating comes from field studies and from numerous experimental studies (cf. Macht, 2008; Renner et al., 2012). The results show that negative affect (NA) typically increases the amount of food intake within restrained eaters. Conversely, within “normal” eaters (i.e., normal weight persons whose emotional and restrained eating scores fall within the normal range), a considerable variability in emotional eating emerged. Macht (2008) reviewed studies on changes in eating in response to emotional stress, including more than 4,700 subjects, and found huge variations in the proportion of participants who reported eating more (4–55%) or less (32–70%) as a response. The experiments included in this review, which examine the impact of negative emotions on eating within normal eaters, showed an increase in food consumption in 43% of the studies, whereas 39% found a decrease and 26% found no effect (Macht, 2008). A comparable inconsistency of the effect of negative emotions on food consumption has also been observed within field studies using self-report. For example, 44% of the participants in a survey study reported that they eat more in response to NA while 48% stated that they eat less (Willenbring et al., 1986). In order to reconsolidate the inconsistent findings, it has been suggested that NA does not uniformly elicit increased food intake but that the way people regulate NA is the key to the triggered eating response (Evers et al., 2010).

Emotion regulation (ER) encompasses efforts through which people alter the experience and/or expression of their emotions (Gross and Thompson, 2007). Conceptually, two different forms of ER strategies can be distinguished: cognitive reappraisal and expressive suppression (Gross and John, 2003). Reappraisal is predominantly an antecedent strategy since it encompasses an evaluation of the meaning of the situation in order to change the emotional impact when the situation occurs. In contrast, suppression is a response strategy as it entails the suppression of behaviors associated with the emotional response during the emotion-triggering situation. The two strategies appear to differ in the required amount of self-regulatory resources. In particular, cognitive reappraisal seems to alter the primary appraisals of emotional stimuli without the need of sustained self-regulatory effort over time (Gross and Levenson, 1993; Richards and Gross, 2006). Conversely, behavioral suppression involves active efforts to inhibit predominant responses, leading to comparably greater “resource depletion” than reappraisal (Baumeister, 2003).

Accordingly, not the fact that people experience negative emotions, but the way in which they cope with them, may determine the emotional impact on eating behavior (Evers et al., 2010). Specifically, the more “costly” an ER is in terms of consuming self-regulatory resources, the more people are likely to be vulnerable to increased food consumption as a secondary regulation strategy. The scarce evidence addressing this issue seems to support the notion that different ER strategies are associated with different patterns of food intake. Vohs and Heatherton (2000) asked dieters to suppress their emotions and showed that they consumed more food than dieters who were asked to express their emotions freely. Thus, suppression seems to be maladaptive in terms of an increased food intake. Conversely, reappraisal appears to be associated with a decrease in food intake, as suggested by Mischel (1996) who showed that reappraisal decreased the rate of immediate food consumption within children. Recently, in a first study, Evers et al. (2010) tested the impact of both regulation strategies within a (bogus) taste test. In line with the resource depletion notion, they found that suppression was associated with a significantly higher amount of comfort food intake in comparison to reappraisal (Study 3). Thus, this study provides first support for the notion that the way people regulate their negative emotions modulates the amount of food intake.

However, a (bogus) taste test paradigm, per definition, requires participants to eat different food items in order to evaluate taste and structure as well as to describe their perception of the food. Assuming that NA does not necessarily trigger eating *per se*, but that inadequate ER causes people to use secondary coping strategies such as eating; in our opinion the question should not focus on “how much people eat when they are asked to eat” but “who begins to eat in the first place”? Thus, we expect that the natural coping sequence is such that when confronted with a negative stimulus that induces NA, people try to regulate it. If they use an effective (or adaptive) ER, the likelihood that they will need to use eating as a secondary ER strategy is comparably low. In contrast, if they use an ineffective ER, it is more likely that they will need to use eating as secondary ER strategy. However, if we explicitly ask people to eat by employing a (bogus) taste test, this crucial first distinction cannot be assessed.

Moreover, in real-life situations, we rarely get the chance to first reappraise a negative event before we are actually confronted with it, as in laboratory settings where participants first receive instructions to reappraise an emotional event and only confront it afterward. In order to test the theoretically assumed sequence (1) negative stimulus, (2) NA, (3) ER, (4) secondary coping response (eating yes/no) in a more ecologically valid way, ER instructions need to be given after the negative event has started to unfold. By allowing the negative emotion to begin to unfold, then providing ER instructions in a time lag manner, the impact of different ERs can be tested while ensuring a comparable “emotional baseline” within a more ecologically valid experimental setting (cf. also Sheppes and Gross, 2011).

## Aims of the Present Study

In order to examine the impact of negative emotions and ER strategies on eating, participants were exposed to two types of comfort food (salty and sweet) after watching a fear-inducing movie. The present study aims were threefold.

First, extending previous research by using a “non-forced” food consumption paradigm, it was tested whether people use eating as a secondary coping strategy when ER is ineffective. Assuming that suppression is a maladaptive ER whereas reappraisal is an adaptive ER, suppression in comparison to reappraisal should lead to an increase in the likelihood of eating.

Second, the non-forced eating setting allows for a clearer pinpointing of the effect of maladaptive ER by providing a distinction between the occurrence of eating (yes/no) and the amount of food consumed as a coping response. Hence, we propose that participants enacting suppression as ER are more likely to eat. According to previous research, suppression should also lead to a greater amount of food consumption as compared to reappraisal.

Third, in order to produce a more ecologically valid experimental setting, the ER instructions were given after the negative event had started to unfold. To our knowledge, this is the first study that employs this procedure in relation to a behavioral outcome.

## Materials and Methods

### Participants

Undergraduate students from Babes-Bolyai University (*N* = 165, 153 women), aged 19–48 years old (*M* = 22.96, SD = 5.44), took part in the study. All participants were of a normal weight range (BMI < 30) with an average BMI of 20.79, SD = 2.84. The mean level of the DEBQ scales (Van Strien et al., 1986), restrained eating (*M* = 2.89, SD = 1.07), emotional eating (*M* = 2.03, SD = 0.89), and external eating (*M* = 2.92, SD = 0.70), were within the normal range. Participants were randomly assigned to one of the three experimental groups defined by ER strategy instruction: cognitive reappraisal (*n* = 58), suppression (*n* = 60), or no ER instruction (*n* = 48).

The experiment was approved by the Ethical Board of Babes-Bolyai University. Also, all participants were informed about the procedures and gave informed consent prior to the experiment.

### Materials and procedure

Participants were informed that the study would investigate the effectiveness of different ER strategies in alleviating negative emotions. In order to ensure reasonably standardized levels of satiety, they were asked to refrain from eating 3 h prior to the experiment, which is a low deprivation level (cf. Schupp and Renner, 2011). Upon their arrival (T1), participants filled in the General NA and Fear scales from the PANAS-X (Watson and Clark, 1999) as a baseline measure of emotion. Moreover, dietary restraint and the tendency to eat more when cognitive restraint of eating is disrupted by psychological, sensory, or emotional challenges were assessed with the Restraint, Emotional, and External Eating scales from the DEBQ (Van Strien et al., 1986). Since the preference for sweet or salty food might differ across participants when in an emotional state (see van Strien, 2010), each participant received two bowls: one filled with potato chips and one with chocolate. Each bowl was filled with 125 g of the respective snack. Participants were told that the food represented an incentive for participation in the study and they were encouraged to feel free to help themselves. The food bowls were presented unobtrusively at the beginning of the procedure in order not to raise suspicions about the real purpose of the experiment. It is important to note that none of the participants ate either the chips or the chocolate while watching the movie clips (during this time the experimenter was present in the room). Thus, the unobtrusive introduction of palpable food was successful because no participant ate before the ER instruction and none of them articulated suspicion about the purpose of the food manipulation.

In a first phase, a 4-min movie clip from “Silence of the Lambs” (Demme, 1991) was shown to reliably elicit fear (Gross and Levenson, 1995) without ER instruction. This allowed participants to become familiar with the experimental setting while ensuring a comparable “emotional baseline” with a more ecologically valid experimental setting.

After watching the first clip, participants were given instructions for ER, following Richards and Gross (2000) standard scripts. They were then informed that they would be shown another movie and instructed to either suppress or reappraise the emotions experienced while watching the clip. The control group, however, received no instruction prior to watching the movie clip. The second clip was a 3 min 30 s scene from “Dancer in the Dark” (Windeløv and Von Trier, 2000) that depicted a violent execution excerpt, shown to reliably elicit high levels of fear in a separate pilot-test (*N* = 10). The reappraisal group were instructed: “*While watching the next movie clip, take a detached, objective perspective. Try to behave like a film director whose task is to assess the performance of each actor. Keep in mind that what you see is purely fictional, it isn*’*t real. You*’*re only a film director and you have to take emotional distance in order to see all the technical details of the scene and actors*’ *performances*.” The suppression instruction was, as follows: “*While watching the next movie clip, try to refrain from showing any feelings you might experience, so that a person looking at you would be clueless regarding your emotions or the type of movie you*’*re* *watching. It is very important not to let your emotions show on your face, in gestures or body movements and not to let the student assistant know how you feel. Try to keep a neutral expression the whole time*.”

Following the second movie clip, the experimenter left the room and participants filled in the post-questionnaires (T2) including the subscales of the PANAS-X, a translated version of the Emotional Regulation Questionnaire (ERQ; Gross and John, 2003), in order to assess enacted emotion regulation strategies, measurements of hunger, and demographic data (gender, age, height, and weight). The items from the ERQ were adapted for the purpose of the present study and participants were instructed to assess the extent to which they used suppression and/or reappraisal while watching the second movie clip. Participants were left alone with the food for about 20–25 min, interval in which they had to fill in the post-questionnaires. None of them left the room earlier. Hence, the time spent in the lab and, as a consequence, the time spent with food/eating did not vary substantially across participants and ER condition. All participants were subsequently debriefed.

The food bowls were weighed at both the beginning and the end of the experiment. An index of food intake (for both food categories) was created by subtracting the final weight from the initial one. For ease of interpretation, all subsequent analyses report food intake in grams (raw scores). Results are reported separately for the two types of food and the total food consumption (sum score) because preferences for sweet and salty food may differ across participants when in an emotional state (cf. van Strien, 2010) or for differences in caloric value.

## Results

### Manipulation check: Induced negative emotion

**Figure 1:**
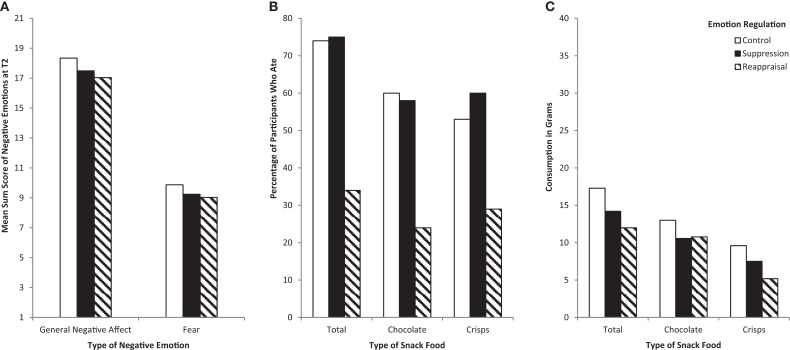
**(A)** Negative emotions (T2, *N* = 165), **(B)** percentage of participants who ate (*N* = 165), **(C)** amount of food consumed (for participants who ate) in the emotion regulation condition.

Experienced fear and NA were subject to a 3 (ER instruction condition: suppression, reappraisal, control) × 2 (time: before vs. after movie clip) ANOVA, with time as a within-subject variable. The main effect of time was significant, indicating that both fear, *F*(1, 156) = 1.50, *p* < 0.001, ${\text{η}}_{p}^{2}=0.90$, and NA, *F*(1, 156) = 99.43, *p* < 0.001, ${\text{η}}_{p}^{2}=0.38$, increased after watching the movie (Fear: *M*_before_ = 7.18, SD* *= 2.39; *M*_after_ = 9.31; SD = 3.95; NA: *M*_before_ = 12.80, SD = 4.06. *M*_after_ = 17.57; SD = 6.61). Neither the main effect for condition, nor the interaction between time and condition were statistically significant, all *F*s < 0.73, *p*s < 0.76, for fear and negative emotions, respectively. Thus, the manipulation was equally successful in inducing a heightened negative emotional state in all three conditions and a comparable negative emotional state was observed across the three ER instruction groups at T2, see also Figure 1A.

Also, no ER instruction condition differences in self-reported hunger *F*(2, 162) = 0.53, *p* = 0.58 (*M* = 2.94, SD = 1.61) or in habitual restrained, emotional, or external eating behavior, all *F*(2, 154) < 1.3, all *p*s < 0.28, were observed.

### Manipulation check: Enacted emotion regulation strategies

Enacted ER strategies (suppression, reappraisal) reported after watching the movie clip were analyzed using a MANOVA with a three-level between subjects factor (ER instruction condition: suppression, reappraisal, control). The multivariate effect for enacted ER strategies was significant with *F*(4, 318) = 4.10, *p *< 0.003, ${\text{η}}_{p}^{2}=0.04$ The three conditions differed in the extent to which they used both suppression, *F*(2, 318) = 4.61, *p* < 0.01, ${\text{η}}_{p}^{2}=0.05$, and reappraisal, *F*(2, 318) = 4.51, *p* < 0.01, ${\text{η}}_{p}^{2}=0.05$, in order to control their emotional experiences. Bonferroni contrast analyses effects showed that participants in the reappraisal group used cognitive reappraisal more than those in the control group (*p* < 0.05) but to the same extent as those in the suppression group (ns.). Conversely, participants in the suppression group used expressive suppression to a larger extend than the reappraisal and the control group (*p*s < 0.05), suggesting that the experimental manipulation was effective.

### Eating vs. non-eating: Impact of emotions and emotion regulation

Overall, 100 out of 165 participants (61%) ate crisps, chocolate, or both, whereas 65 participants (39%) did not eat from the offered food samples. No difference between crisps and chocolate consumption was found: 22 participants (13%) ate only crisps, 22 participants (13%) ate only chocolate, and 55 participants (33%) ate both.

To examine whether the amount of negative emotion experienced predicted whether participants ate or not, two separate logistic regression analyses were conducted with fear and NA scores (at T2) as predictors of eating status (eating vs. non-eating). The results show that neither fear, nor NA at T2 predicted eating vs. non-eating. This holds true for total food, β = −0.02, *p* > 0.35, sweet snacks (chocolate), β = −0.02, *p* > 0.28, and salty snacks (crisps), β = −0.03, *p *> 0.17, respectively. Likewise, changes in fear and NA (T1–T2) did not predict eating vs. non-eating, all βs < 0.06, *p*s > 0.10, for total food, chocolate, and crisps.

In order to test the effect of the different ER instruction on eating (eating vs. non-eating), a chi square test of independence was performed yielding a significant effect for the ER condition with χ^2^(2, *N* = 165) = 25.56, *p* < 0.001. As Figure 1B shows, 75% of the participants in the suppression condition and 74.5% in the control condition started to eat. Conversely, only 34.5% of the reappraisal condition ate from the sweet and salty snacks. A similar pattern of result was also found for the sweet snacks [χ^2^(2, *N* = 165) = 18.25, *p* < 0.001] and for the salty snacks [χ^2^(2, *N* = 165) = 12.07, *p* = 0.002].

#### Control analyses

In order to ensure the reliability of the present results, additional control analyses were conducted. In a first step, it was tested whether the observed effect of the ER instruction condition was due to differences in the habitual eating patterns. Therefore, a logistic regression analysis was conducted with the dichotomous dependent variable “eating status” (eating vs. non-eating) and “ER instruction condition” (suppression, reappraisal, control) and the three habitual eating scales (DEBQ-Restraint, DEBQ-Emotional, and DEBQ-External Eating) as predictors. Again, “ER instruction condition” was a significant predictor (β < −0.88, *p* < 0.001), whereas none of the three habitual eating patterns were statistically significant, all βs < −0.02, *p*s > 0.55. Thus, habitual eating such as restrained eating, emotional eating, or external eating did not predict who started to eat or who refrained from eating.

In a second step, the impact of the self-reported amount of ER was tested. A logistic regression was conducted with eating status (eating vs. non-eating) as the dependent variable and “ER instruction condition” (suppression, reappraisal, control), “self-reported suppression,” and “self-reported reappraisal” as predictors. Replicating previously reported results, the logistic regression yielded a significant effect for the “ER instruction condition” with (β < −0.89, *p* < 0.001). However, the self-reported amount of suppression and reappraisal did not contribute to explaining additional variance, all βs < −0.07, *p*s > 0.12.

### Amount of food intake: Impact of emotions and emotion regulation

In subsequent analyses, the amount of food consumed was examined. In line with previous research, the amount of consumed food was analyzed across all participants, irrespective of whether they ate or not. Again, a significant main effect of the factor “ER instruction condition” (suppression, reappraisal, control) was found for all three different food amount scores: total food *F*(2, 162) = 5.24, *p *< 0.001, chocolate, *F*(2, 162) = 3.10, *p *< 0.05, and crisps *F*(2, 162) = 4.59, *p* < 0.01. As Bonferroni *post hoc* analyses showed, reappraisers ate significantly less food in total (*M* = 4.13, SD = 9.06) and less crisps (*M* = 1.53, SD = 3.15) than either the suppressors (total amount: *M* = 10.56, SD = 12.31; crisps: *M* = 4.48, SD = 6.25) or the control group (total amount: *M* = 12.84, SD = 21.31; crisps: *M* = 5.12, SD = 9.60), *p*s < 0.05. Moreover, they ate less chocolate (*M* = 2.60, SD = 7.65) as compared to the control group (*M* = 6.16, SD = 9.48), *p* = 0.05.

Extending previous research, the impact of emotions and regulation strategies on the amount of food intake in grams was analyzed for participants who began to eat each respective food item (see also Figure 1C). In contrast to previous research, participants were free to eat or not to eat in the present *ad libitum* food setting. Thus, analyzing only participants (*n* = 100) who actually began to eat provides more precise information about the actual amount of consumption.

In a first step, multiple regressions analyses were conducted to examine the impact of General NA and fear (at T2) on the amount of consumed food (total food, chocolate, crisps). The results show that neither General NA (T2) nor fear (T2) predicted the amount of food intake, with all βs < |0.69|, *p*s > 0.13 for total food, chocolate, and crisps. Likewise, neither changes in General NA from T1 to T2 nor changes in fear predicted the total food, chocolate, or crisps intake, with all βs* *< |0.48|, *p*s* *> 0.22.

In a second step, the impact of ER instruction on food intake was examined. Three ANOVAs with the dependent variable observed amount of food intake in grams and the three-level factor “ER instruction condition” (suppression, reappraisal, control) were conducted. No significant effect for the factor “ER instruction condition” emerged: total food intake, *F*(2, 97) = 0.679, *p* = 0.509; chocolate *F*(2, 74) = 0.25, *p* = 0.77; crisps *F*(2, 75) = 1.52, *p* = 0.22. Thus, given that participants began to eat, they consumed a comparable amount of food irrespective of the ER instruction condition or the type of available comfort food.

#### Control analyses

Additional ANCOVAs were conducted as control analyses with the three different measures of the amount of food intake (total, chocolate, crisps) as dependent variables, respectively, and the between subjects factor “ER instruction condition” (suppression, reappraisal, control). Using the three different habitual eating patterns as additional covariates yielded a virtually unchanged pattern of results: with a non-significant factor “ER instruction condition,” total food intake measured *F*(2, 76) = 0.79, *p* = 0.45; chocolate, *F*(2, 56) = 0.32, *p* = 0.54; crisps, *F*(2,57) = 0.87, *p* = 0.42; and the non-significant effects for the three covariates with all *t*s < 1.50, *p*s > 0.14. Similarly, the self-reported amount of ER, as well as age, gender, or BMI as covariates were statistically non-significant.

## Discussion

Research has recently turned to studying individual differences in ER as a predictor of emotional eating (Evers et al., 2010). With the present study, we extended previous research by allowing participants to choose not only how much they wanted to eat but also whether they wanted to eat at all (*ad libitum* food intake). Thus, the present study differentiated between two core questions: “Who begins to eat?” and “How much do people eat when they start to eat?” Also, this study is the first to investigate whether there are differences in the effectiveness between the two ER strategies (suppression, reappraisal) when they are employed at the same point in the unfolding of the emotional response.

Focusing on the question “Who began to eat?” the present results clearly show that participants in the reappraisal group were less likely to eat both chocolate and crisps, compared to the control and suppression groups. Among the reappraisal group, only 1/3 started to eat, whereas 3/4 among the suppression group and 3/4 among the control group started to eat. Thus, reappraising but expressing negative emotions seems to be a highly effective regulation strategy; whereas, suppression appears to be rather ineffective.

Consequently, across the total sample, including both participants who started to eat and those who did not, the amount of consumed food differed greatly in dependence of the ER condition. Within the reappraisal group, the amount of consumed food was on average significantly lower than in the suppression or control groups. On average, reappraisers ate 61% less than suppressors and 68% less than the control group. These results are consistent with findings from related studies suggesting that reappraisal, in comparison to suppression, is associated with reduced food intake in women (e.g., Evers et al., 2010) and a reduced desire to binge in women with binge eating disorder (Svaldi et al., 2010).

However, a greatly different picture emerged when the impact of ER strategies on the amount of consumed food was analyzed for participants who actually ate from the respective food item. In contrast to previous research, participants were free to eat or not to eat in the present *ad libitum* food setting, thus allowing for the distinction between the question “who begins to eat?” and the question “how much do people eat, when they start to eat?” The present results show that reappraisers ate as much as participants in the suppression group or in the control group once they had begun to eat. Thus, the main difference between the three ER conditions seems to be whether eating is employed as a secondary regulation strategy at all rather than the amount of food needed for secondary regulation as suggested in previous research (e.g., Evers et al., 2010).

Interestingly, reappraisers did not show a lower level or increase in NA or fear compared to the suppressors or the control group. The comparable level or increase in negative emotions across all three emotions regulation conditions was probably due to the timing of ER instruction. By giving the ER instructions after the negative event had started to unfold, we aimed at improving the ecological validity of the study. Specifically, the sequence (1) negative stimulus, (2) NA, (3) ER instruction, (4) secondary coping response (eating yes/no) reflects real-life situations in a more ecologically valid way since we rarely have the chance to reappraise a negative emotional event before we actually deal with it. However, one could argue that at higher levels of emotional intensity, the differences in effectiveness between the reappraisal and suppression might be blurred (Sheppes and Gross, 2011). Thus, only using ER for the second movie clip might have caused an intensification of negative emotions in all conditions. Reappraisal, employed relatively late in the emotion iterative phase was weakened, given that most of the cognitive resources were allocated to managing the unfolding emotion. Though reappraisal was not effective in alleviating negative emotions, reappraisal was nonetheless more “sparing” in terms of resource allocation, allowing participants to better regulate food intake.

Hence, the results suggest that the advantage of reappraisal is that people have the same emotional outcome (increase in NA) but with less reliance on maladaptive secondary regulation strategies such as eating, compared to suppressors or the control group. Therefore, the total “net profit” is more favorable for reappraisal than for suppression or spontaneous emotions regulation since less reliance on secondary maladaptive coping is required in order to arrive at the same emotional outcome. One might speculate that suppression is a comparably costly regulation strategy that leads to more rapid self-regulation depletion, which is in turn supplemented by secondary regulation strategies such as eating. However, this is speculative and future studies might trace the course of eating (e.g., delay in eating, pace of eating).

In the present study, the control group, which received no ER instruction, behaved in a highly similar way to the suppression group. Both groups were more likely to eat than the reappraisal group. Consequently, both groups consumed more food when all participants, irrespective of their eating status (eating vs. non-eating), were included in the analyses. Conversely, in the study conducted by Evers et al. (2010 the control group ate less than the suppression group. We would like to argue that these inconsistent findings reflect the puzzling inconsistency previously reported in empirical studies examining the effect of negative emotions on eating within normal eaters (cf. Macht, 2008). Specifically, since the control condition received no regulatory instruction, the strategies employed depend on the respective sample and might differ between the studies, resulting in the observed inconsistent results.

All participants were asked to refrain from eating for the 3 h prior to the experiment. Thus, food might become more attractive to participants and eating might had become the “default” option or right strategy. In general, 3 h of non-eating is commonly considered as a mild form of “deprivation” precluding that participants are completely satiated (Oliver et al., 2000; Schupp and Renner, 2011). Moreover, assuming that eating becomes the “default” option after 3 h of non-eating, we should have found a general increase in the frequency of eating across groups. However, we found a pronounced differential eating pattern independent of the ER strategy, rendering a “default option” rather unlikely. Moreover, there was neither a difference in hunger levels between groups nor in the elapsed time prior to eating. Accordingly, we would like to argue that eating in the present context was mainly a secondary coping reaction.

However, the type of ER is but one factor contributing to emotional eating. One might speculate that a high tendency to regulate NA by eating can be counteracted not only by ER but also by a high capacity of self-control (Sproesser et al., 2011). Overall, general self-control seems to be an important moderator of food choice and healthy diet, opposing emotional eating, and influencing the extent to which foods are considered tempting (de Ridder and de Wit, 2006).

As the manipulation check showed, there might be some co-activation of ER strategies (Szasz et al., 2011). ER is employed spontaneously when an emotional encounter occurs, even in the absence of specific instructions (Gross, 2008); therefore individual preferences for certain types of ER can interfere with ER instructions. However, our results confirmed that participants used the ER strategies they were encouraged to use, even though they mainly employed other ER strategies spontaneously.

Despite these limitations, our results are innovative in two ways. First, they show that different ER strategies are associated with different odds of eating in the first place. Secondly, even when reappraisal is employed relatively late in the emotional process, it is still associated with a lower rate and a lower amount of food intake, although it is not as beneficial for reducing negative emotions. Thus, the main difference between the two ER strategies, suppression and reappraisal, is whether or not eating is needed as a secondary coping strategy, rather than differences in the amount of consumed food per person as suggested in the study by Evers et al. 2010).

All in all, this study adds to the growing body of evidence, showing that the combination of ER strategies might be crucial for adaptive ER. Moreover, it seems to be pertinent to take the “total net outcome” into account in terms of invested self-regulatory resources and behavioral outcome. In the face of negative events, successful reappraisers who show a low level of suppression, might feel as threatened and scared as suppressors but require less high caloric “fuel” to arrive at the same emotional condition.

## Conflict of Interest Statement

The authors declare that the research was conducted in the absence of any commercial or financial relationships that could be construed as a potential conflict of interest.
